# Barriers to Engage Low-Skilled Adults in Educational Opportunities: A Global Perspective

**DOI:** 10.1093/geroni/igaa057.208

**Published:** 2020-12-16

**Authors:** Abigail Helsinger, Nytasia Hicks, Meghan Young, Oksana Dikhtyar, Phyllis Cummins, Taka Yamashita

**Affiliations:** 1 Miami University, Oxford, Ohio, United States; 2 University of Maryland, Baltimore, Maryland, United States

## Abstract

The demand for adult education and training (AET) opportunities is substantial as older adults are remaining in the labor force at older ages, and are facing substantial technological changes in the workplace. Strategies to engage middle-aged and older adult workers in AET often exclude low-skilled and sub-populations. The engagement of these sub-populations in AET is challenging as access, awareness, and program costs associated with AET opportunities often target highly skilled populations. The inequality in AET participation warrants specific programs and strategies to address challenges low-skilled adult workers face in pursuing AET. The purpose of this study is to identify AET opportunities for low-skilled middle-aged and older adults, as well as highlight major barriers to engage and retain these sub-population in AET. Data were collected from 36 key informants through semi-structured interviews and through document reviews. Key informants represented Australia, Canada, Italy, Norway, the Netherlands, the U.K., and the U.S. Descriptive methods were used to identify barriers in recruiting and retaining low-skilled middle-aged and older adults. We particularly focused on the barriers related to cost, language, access, and awareness. Results highlighted opportunities tailored to support adult workers in the pursuit of adult learning opportunities both domestically and internationally. Barriers including learning histories, lack of long-term person-centered support, as well as the role of multiple forms of learning, such as formal and informal learning, were identified. Last, we provide recommendations for recruiting and retaining middle-aged and older adult workers in AET programs.

